# Young Old People

**Published:** 1859-06

**Authors:** 


### YOUNG OLD PEOPLE.

Some look old at less than forty, others beyond threescore have the vivacity, the sprightliness, and the spring of youth. One of the most active politicians of the times is now in his seventy-fifth year, and yet goes by the name of "the ever youthful Palmerston." who with the weight of nations on his shoulders, would find time to take a rapid ride on horseback every day, from ten to twenty miles. "The heavy cares and severe labors of the Earl of Malmesbury average eleven hours a day," and yet at the age of "fifty years, is scarcely above forty in appearance." It is by no means an uncommon thing to read the deaths of men and women of the English nobility at eighty and ninety years, to be accounted for in the fact of their taking time to do things, and thereby doubling the time for doing them. The British are a dignified people, manly, mature; a deliberative people, with the result of being as a nation, the most solid, the most substantial, and the greatest on the globe. They are worthy of that greatness, and we above all the peoples should be proud of it. Americans on the other hand are a hasty race; their habitual hurries and anxieties eat out the very essence of life before half that life is done, and all bloodless, fidgetty, skinny and thin, we are but "a vapor that appeareth for a little time, and then vanisheth away."

